# Uncommon Causes of Interlobular Septal Thickening on CT Images and Their Distinguishing Features

**DOI:** 10.3390/tomography10040045

**Published:** 2024-04-17

**Authors:** Achala Donuru, Drew A. Torigian, Friedrich Knollmann

**Affiliations:** Division of Cardiothoracic Imaging, Department of Radiology, Hospitals of University of Pennsylvania, Philadelphia, PA 19104, USA; drew.torigian@pennmedicine.upenn.edu (D.A.T.); friedrich.knollmann@pennmedicine.upenn.edu (F.K.)

**Keywords:** interlobular septal thickening (ILST), computed tomography (CT), ground glass opacity (GGO), differential diagnosis

## Abstract

Interlobular septa thickening (ILST) is a common and easily recognized feature on computed tomography (CT) images in many lung disorders. ILST thickening can be smooth (most common), nodular, or irregular. Smooth ILST can be seen in pulmonary edema, pulmonary alveolar proteinosis, and lymphangitic spread of tumors. Nodular ILST can be seen in the lymphangitic spread of tumors, sarcoidosis, and silicosis. Irregular ILST is a finding suggestive of interstitial fibrosis, which is a common finding in fibrotic lung diseases, including sarcoidosis and usual interstitial pneumonia. Pulmonary edema and lymphangitic spread of tumors are the commonly encountered causes of ILST. It is important to narrow down the differential diagnosis as much as possible by assessing the appearance and distribution of ILST, as well as other pulmonary and extrapulmonary findings. This review will focus on the CT characterization of the secondary pulmonary lobule and ILST. Various uncommon causes of ILST will be discussed, including infections, interstitial pneumonia, depositional/infiltrative conditions, inhalational disorders, malignancies, congenital/inherited conditions, and iatrogenic causes. Awareness of the imaging appearance and various causes of ILST allows for a systematic approach, which is important for a timely diagnosis. This study highlights the importance of a structured approach to CT scan analysis that considers ILST characteristics, associated findings, and differential diagnostic considerations to facilitate accurate diagnoses.

## 1. Introduction

Interlobular septa are the thin connective tissue margins demarcating secondary pulmonary lobules within the lung parenchyma. While typically invisible on high-resolution computed tomography (HRCT) scans, these septa can become thickened in various disease processes. Low-dose ultra-high-resolution computed tomography (CT) of the lungs using photon-counting CT (PCCT) has shown improved conspicuity of pulmonary pathologies including interlobular septal thickening (ILST) [1].

Anatomy of the secondary pulmonary lobule:

The secondary pulmonary lobule is the fundamental structural unit of the lung parenchyma surrounded by a connective tissue sheath. It is polyhedral or pyramidal in shape, can range from 5 to 25 mm in diameter, and may contain 3–25 acini. Understanding its structure is crucial for interpreting ILST on CT scans because different pathologies can affect various components of the lobule.

Key components:

- Centrilobular region: Located centrally, it contains pre-terminal, terminal, and respiratory bronchioles and accompanying pulmonary arterioles and lymphatic vessels.
- Lobular parenchyma: This is the bulk of the secondary pulmonary lobule, surrounding the centrilobular region. It consists of the following:
  ◦ Respiratory acini: These microscopic units are the functional sites of gas exchange.
  ◦ Intralobular septa: These are delicate connective tissue structures within the secondary pulmonary lobule that support the alveoli and house the capillary network. They are not typically visible on CT scans of healthy patients.
- Interlobular septa: These are thicker connective tissue structures along the periphery of secondary pulmonary lobules that are perpendicularly oriented to the pleura. They contain lymphatic vessels and pulmonary venules. ILST refers to the thickening of these interlobular septa, which can be seen on CT scans, although isolated interlobular septa may occasionally be visible on CT in healthy patients in the lung apices and bases [2,3].

Thickening of the interlobular septa is a common and easily recognized CT feature of many lung disorders. Various entities that are uncommonly associated with ILST are listed in Table 1. ILST can be smooth, nodular, or irregular. Smooth ILST is the most common of the three patterns and the least specific, and it can be found in a large number of venous, lymphatic, and infiltrative disorders. Nodular ILST is a finding that is associated with a more specific group of disorders. Irregular ILST is seen in patients with underlying architectural distortion of the lungs, namely due to fibrotic lung disease. ILST is an uncommon finding in amyloidosis, sarcoidosis, lymphoproliferative disorders (lymphomas and lymphoid interstitial pneumonia), and silicosis (Table 2).

The most common cause of ILST is congestive heart failure (CHF), but there are other non-cardiogenic causes. In CHF, ILST is typically diffuse, bilateral, and associated with increased attenuation of the lung parenchyma. Septal thickening on CT images typically occurs when the pulmonary capillary wedge pressure (PCWP) reaches 20–25 mmHg, but the exact PCWP at which it appears can vary depending on a number of factors such as the patient’s age and underlying medical conditions [2]. The presence of cardiomegaly, bilateral pleural effusions, and typical clinical presentation findings aid with the diagnosis. The causes of ILST and their imaging features are summarized (Table 3). The distinctive clinical features of various diagnoses discussed in this manuscript and associated extrapulmonary findings are discussed (Table 4). As pulmonary edema and lymphangitic spread of tumors are commonly encountered conditions in routine clinical practice, these will not otherwise be discussed in this manuscript.

CT has higher sensitivity than chest radiography (CXR) for detecting septal thickening. HRCT is superior to conventional CT in the morphologic delineation of pulmonary parenchyma [4]. The most common reconstruction algorithms used for HRCT exams are filtered back projection (FBP) and iterative reconstruction (IR). The recommended slice thickness for HRCT exams is 1–1.5 mm. Studies have demonstrated that high matrix image reconstruction significantly improves the visualization of imaging features characteristic of interstitial lung disease (ILD) [5].

## 2. Infections

### 2.1. COVID-19 Pneumonia

**Figure 1:**
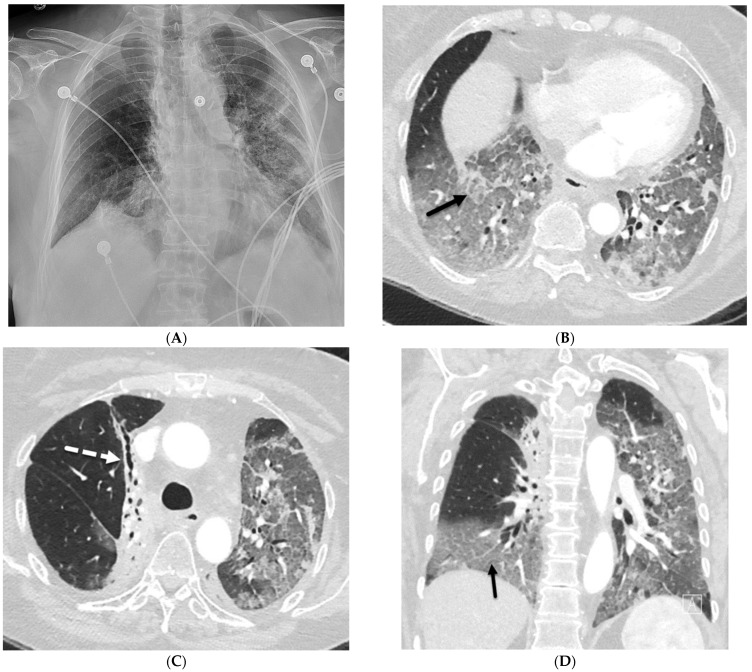
A 61-year-old female with a history of metastatic non-small-cell lung cancer presents with acute hypoxic respiratory failure due to COVID-19 pneumonia. A frontal chest radiograph (**A**) demonstrates interstitial prominence in the left lung. Axial (**B**,**C**) and coronal (**D**) chest CT images demonstrate radiation fibrosis in the right perihilar region (dashed white arrow). Diffuse ground glass opacities are noted throughout the left lung and right lung base with interlobular septal thickening (solid black arrows).

While peripherally distributed ground glass opacities (GGOs) are the most common manifestation of COVID-19 pneumonia, consolidation and reticular opacities are also commonly noted. ILST is common in patients with severe COVID-19 pneumonia [6] (Figure 1).

### 2.2. Bacterial Pneumonia

ILST is a frequent finding in bacterial pneumonia and is due to the buildup of inflammatory cells, including neutrophils, lymphocytes, and macrophages, and fluid in the bronchovascular interstitium. Streptococcus pneumonia is the leading cause of community-acquired pneumonia, accounting for approximately 30–50% of cases worldwide. Lobar pneumonia predominantly involving the lower lobes is the most common imaging appearance. Staphylococcus aureus demonstrates consolidation with or without cavitation, centrilobular nodules, and bronchial wall thickening on CT. Centrilobular nodules, GGOs, and lobular consolidation with bronchial wall thickening are common CT findings in Mycoplasma pneumoniae pneumonia. Legionnaires’ disease, caused by Legionella pneumophila shows multilobar airspace disease interspersed with GGO on CT. Klebsiella pneumonia typically presents with homogeneous parenchymal consolidation containing air bronchograms, most prominently in the right upper lobe. Bacterial pneumonia such as Streptococcus Pneumoniae pneumonia can demonstrate ILST on CT [7]. Pulmonary nocardiosis can manifest on CT with a variety of nonspecific findings, including nodules/masses, GGOs, ILST, cavitation, and pleural effusion [8].

### 2.3. Viral Pneumonia

**Figure 2:**
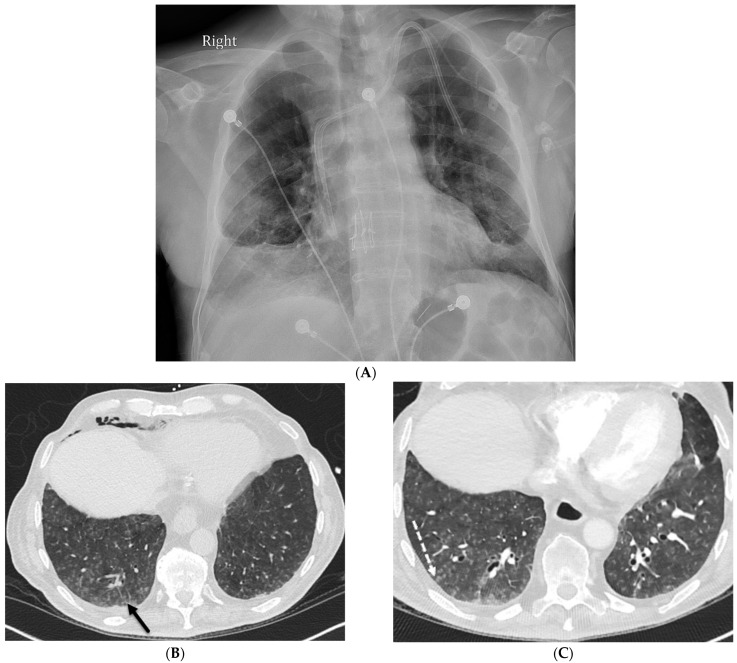
A 68-year-old male with a history of idiopathic pulmonary fibrosis underwent bilateral lung transplantation and now presents with shortness of breath and hypoxia five months later. Bronchoscopy revealed adenovirus infection. The frontal chest on the right in (**A**) demonstrates interstitial prominence. Axial chest CT images (**B**,**C**) reveal tree-in-bud opacities (dashed white arrow) in both lower lobes and smooth interlobular septal thickening (solid black arrow).

ILST is a common finding in viral pneumonia, particularly for cytomegalovirus, influenza A virus, rhinovirus, herpes simplex virus, varicella zoster virus, and human bocavirus pneumonia. Adenovirus pneumonia shows multifocal GGOs with patchy consolidations bilaterally and may show lobar or segmental distribution. In herpes simplex virus pneumonia, predominantly multifocal segmental or subsegmental areas of GGOs and less dominant focal areas of consolidation are noted on CT. In varicella zoster virus infection, CT usually shows 1–10 mm nodules with a surrounding halo of GGOs, patchy GGOs, and coalescence of nodules diffusely throughout both lungs. Bilateral asymmetric GGOs, small centrilobular nodules, and airspace consolidation are the predominant CT findings with cytomegalovirus infection. Mediastinal lymphadenopathy, interstitial infiltrates, and widespread GGOs can be seen with Epstein–Barr virus infection [9] (Figure 2).

### 2.4. Pneumocystis Jiroveci Pneumonia (PJP)

**Figure 3:**
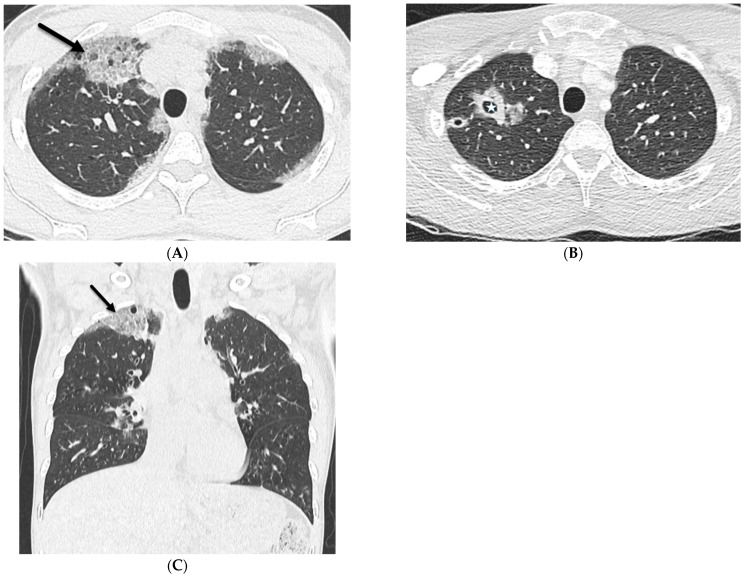
A 32-year-old female with a history of HIV infection presents with a progressive cough and fever. Axial (**A**,**B**) and coronal (**C**) chest CT images demonstrate areas of interlobular septal thickening (solid black arrow) with intralobular lines and interspersed ground glass opacity giving a crazy paving appearance primarily in the upper lung fields. Thin-walled cysts are noted in the right upper lobe compatible with pneumatoceles (star).

PJP pneumonia is most common in people with human immunodeficiency virus (HIV)/acquired immunodeficiency syndrome (AIDS), but it can also occur in those with other conditions that weaken the immune system such as cancer, autoimmune diseases, and long-term use of corticosteroids or other immunosuppressive drugs. HRCT shows extensive GGOs as the primary finding in PJP. In more advanced cases of PJP, septal lines and intralobular lines superimposed on GGOs, also known as crazy paving, and consolidation may develop. Lung consolidation is more common in patients without HIV infection and tends to develop more rapidly, reflecting pulmonary damage from the host immune response. Up to one-third of patients with PJP develop pulmonary cysts of varying shape, size, and wall thickness. Pulmonary cysts are associated with an increased risk of spontaneous pneumothorax [10] (Figure 3).

## 3. Interstitial Lung Diseases

### 3.1. Usual Interstitial Pneumonia (UIP)

The UIP pattern of ILD can be seen in idiopathic pulmonary fibrosis or secondary to underlying systemic diseases such as connective tissue diseases, drug toxicity, asbestosis, and chronic hypersensitivity pneumonitis. ILST, intralobular interstitial thickening, traction bronchiectasis, and honeycombing with peripheral and basilar predominance in the lungs are characteristic CT findings of UIP [11,12]. The ILST can be smooth, nodular, or irregular.

### 3.2. Nonspecific Interstitial Pneumonia (NSIP)

**Figure 4:**
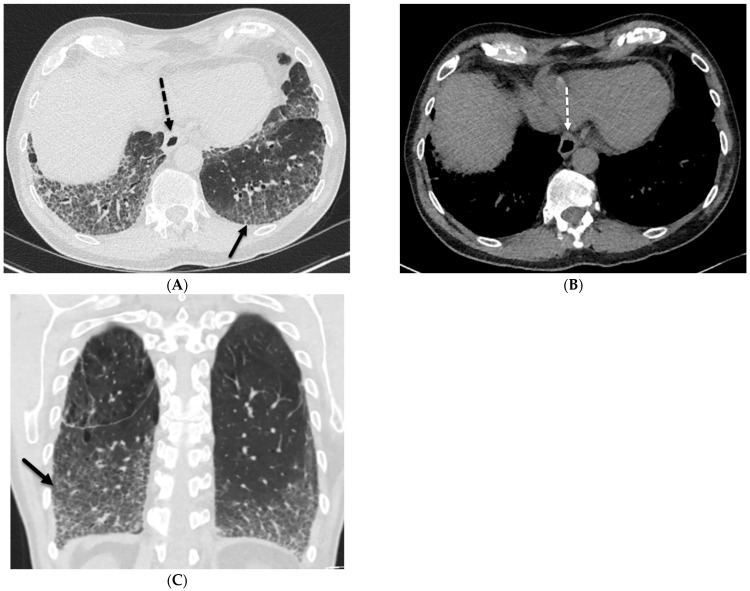
A 62-year-old male with a history of systemic sclerosis. Axial (**A**,**B**) and coronal (**C**) chest CT images demonstrate interstitial fibrotic process characterized by basilar predominant interlobular (solid black arrow) and intralobular septal thickening, traction bronchiectasis, and mild diffuse ground glass opacity without honeycombing. A dilated esophagus (dashed arrow) was also observed consistent with esophageal dysmotility, a common feature of scleroderma.

NSIP is a common ILD that is associated with a number of conditions such as connective tissue disorders including systemic sclerosis (scleroderma), Sjögren’s syndrome, polymyositis, dermatomyositis, and systemic lupus erythematosus, and may sometimes be idiopathic. GGOs and irregular septal lines predominantly in the lower third of the lungs with subpleural sparing can be observed. Associated esophageal dilation may be seen in patients with systemic sclerosis (Figure 4). The diagnosis of NSIP is made based on a combination of clinical findings, imaging studies, and lung biopsy [13].

### 3.3. Hypersensitivity Pneumonitis (HP)

**Figure 5:**
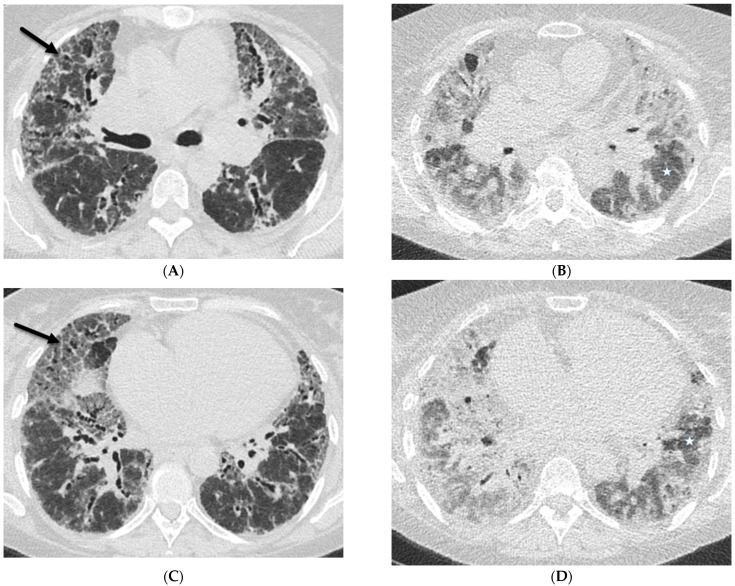
A 60-year-old female with a history of chronic hypersensitivity pneumonitis presents with worsening hypoxemia. Axial inspiratory (**A**,**C**) and expiratory (**B**,**D**) chest CT images demonstrate areas of interlobular septal thickening (solid black arrows), ground glass opacity, and traction bronchiectasis. There is also heterogeneous pulmonary air trapping on the expiratory images (stars).

In acute HP, symmetric upper lung-predominant poorly defined centrilobular nodules are noted. Mid and lower lung zone-predominant GGOs and consolidation can be seen. Superimposed ILST may be present due to areas of pulmonary hemorrhage or edema. In chronic HP, the most common findings are traction bronchiectasis, ILST, and intralobular reticulation, with a distribution that is mainly peribronchovascular without areas of predominance [14]. In fibrotic HP, CT sometimes shows a tortoise shell or a hexagonal pattern which is created by the alternating bands of thickened and normal interlobular septa [15] (Figure 5).

### 3.4. Organizing Pneumonia (OP)

Primary cryptogenic OP occurs without a specific etiology. Secondary OP can be associated with infections, toxins such as smoking and radiation, medications, connective tissue diseases, and lung cancer. In a study by Faria et al., the most common CT finding of OP was that of GGOs and consolidation followed by reticulation, bronchiectasis, interstitial nodules, ILST, perilobular distribution pattern, reverse halo sign (central GGOs with peripheral consolidation), airspace nodules, and halo sign (peripheral GGOs with central solid nodule or consolidation). The lung areas involved were predominantly bilateral, with the middle and lower thirds of the lungs most commonly affected [16].

### 3.5. Lymphoid Interstitial Pneumonia (LIP)

**Figure 6:**
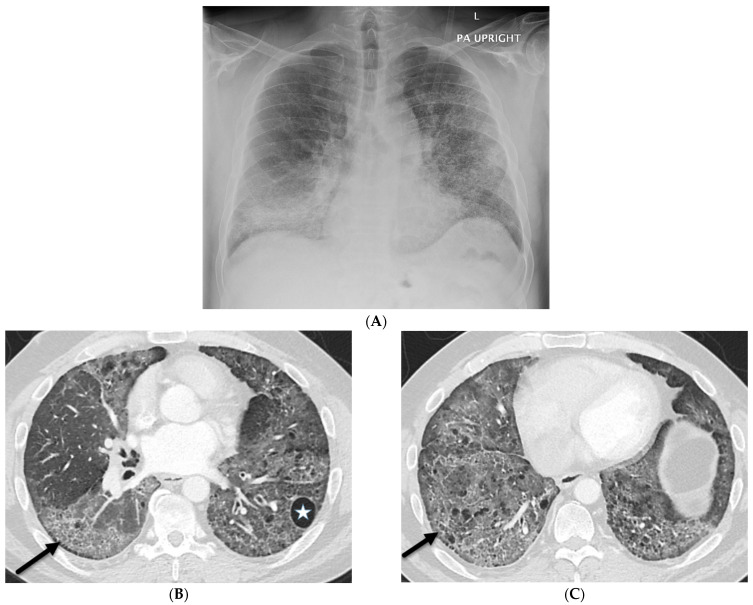
A 47-year-old male with a history of scleroderma and positive ANA titers presents with a cough. A frontal chest radiograph (**A**) demonstrates interstitial prominence in both lungs, the left greater than the right. Axial chest CT images (**B**,**C**) demonstrate widespread basilar predominant ground glass opacities, interlobular septal thickening (solid black arrows), and numerous cysts (star).

LIP is a rare form of ILD usually associated with other systemic diseases such as primary Sjögren’s syndrome, rheumatoid arthritis, systemic lupus erythematosus, mixed connective tissue disease, HIV infection, and some medications such as amiodarone. CT findings of LIP include GGOs, air cysts, ILST, thickening of the peribronchovascular interstitium, and poorly defined centrilobular or subpleural nodules (Figure 6). ILST and intralobular reticular opacities correspond to lymphocytic infiltration and mild fibrosis of interlobular or intralobular septa [17].

## 4. Iatrogenic

### 4.1. Lipoid Pneumonia

**Figure 7:**
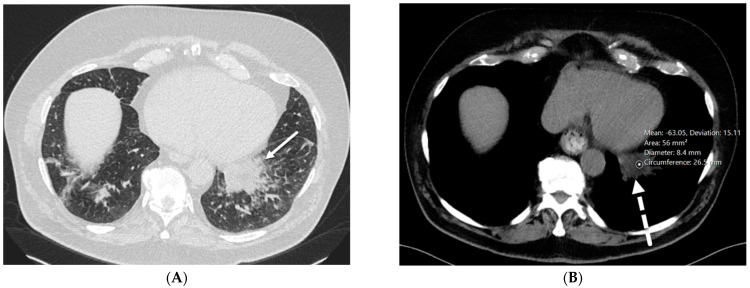
A 76-year-old male with chronic left lower lobe opacities due to lipoid pneumonia. The patient has a prior history of mineral oil ingestion for constipation. Axial chest CT images ((**A**) at baseline and (**B**) two years later) show interlobular septal thickening (solid white arrow) and fat attenuation (−40 to −60 HU) consolidation (dashed white arrow) in the left lower lobe without gross change.

Lipoid pneumonia occurs when lipids accumulate in the lung tissue. It can be exogenous when coming from outside sources such as mineral oil, animal fat, or vegetable oil that are aspirated into the lungs. Endogenous lipoid pneumonia can come from lipid-rich debris from damaged alveolar cells and can be associated with lung cancer or chronic inflammatory lung disorders. The chest radiographic findings of lipoid pneumonia are nonspecific and can include consolidation, nodules, or masses. On CT, the lipoid pneumonia pattern typically shows lower lobe-predominant consolidation with fat attenuation (typically less than −30 HU) and GGOs with or without ILST [18] (Figure 7). Lipid-laden macrophages are often seen in histopathological samples obtained by transthoracic needle biopsy [18].

### 4.2. Drug-Induced Pneumonitis

In a study by Akira et al., ILST and centrilobular opacities are observed in 85% of cases of antibiotic agent-induced pneumonitis and in 88% of cases of non-neoplastic agent-induced pneumonitis. Antimicrobial drug-induced pneumonitis is well reported [19]. It can present with both ILST and GGOs, giving a crazy paving appearance. In drug-induced pneumonitis, the pulmonary interstitium contains eosinophils and other inflammatory cells with desquamation into the alveolar spaces [20]. Drug-induced pneumonitis can be associated with various patterns on CT including OP, NSIP, UIP, HP, diffuse alveolar damage (DAD), acute interstitial pneumonia (AIP), eosinophilic lung disease, and sarcoid-like reactions.

### 4.3. Immunotherapy-Related Side Effects

**Figure 8:**
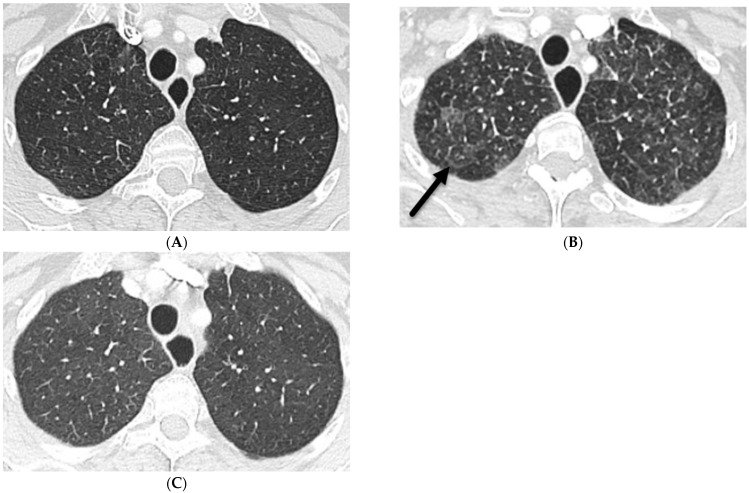
A 68-year-old female with a history of peritoneal mesothelioma. An axial chest CT image at baseline (**A**) shows clear lungs. An axial chest CT image 2 weeks after starting immunotherapy (**B**) demonstrates GGO, perilymphatic nodules, interlobular septal thickening (solid black arrow), and mediastinal and hilar lymphadenopathy (not shown) mimicking sarcoidosis. An axial chest CT image 2 months later after drug discontinuation corticosteroid treatment (**C**) shows near-complete resolution of pulmonary abnormalities.

Immune checkpoint inhibitor administration is associated with specific side effects that most frequently arise during the first months after treatment initiation, although cases after therapy discontinuation have also been reported. Commonly encountered imaging patterns include those of OP, NSIP, HP, AIP, and sarcoid-like reactions. The AIP pattern is characterized by geographic or diffuse GGO or consolidative opacities involving the majority of the lungs, typically with rapid progression. Imaging features of sarcoid-like reaction are similar to those of sarcoidosis and include mediastinal and hilar lymphadenopathy and pulmonary nodules in a perilymphatic distribution, with upper lung predominance (Figure 8). ILST can also be identified with AIP and sarcoid-like reactions [21].

### 4.4. Electronic Cigarette or Vaping Use-Associated Lung Injury (EVALI)

**Figure 9:**
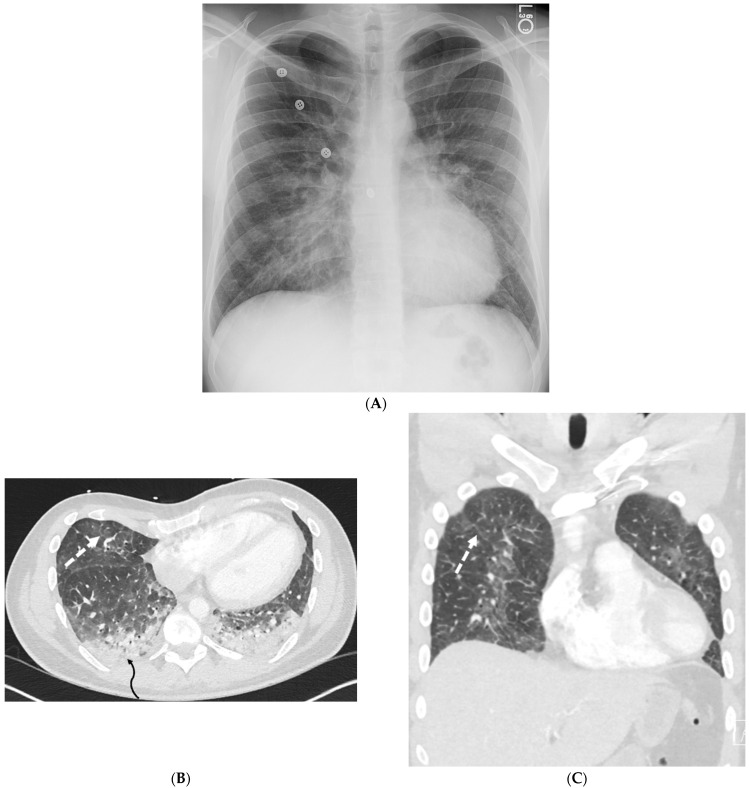
A 25-year-old male with no significant past medical history admitted with acute hypoxic respiratory failure with cough, nausea, and vomiting after vaping one month ago. A frontal chest radiograph (**A**) and axial and coronal chest CT images (**B**,**C**) show diffuse ground glass opacities in the lungs and dependent consolidation (curved black arrow) in bilateral lower lobes. Smooth interlobular septal thickening (dashed white arrow), predominantly in lower lobes, is also visualized on CT images.

EVALI occurs secondary to the use of e-cigarettes or vaping products that contain nicotine or cannabinoids. EVALI manifests on CT as multifocal GGOs, often with consolidation and a small centrilobular nodular pattern resembling HP. Other findings on chest CT include consolidation, nodules, ILST, intralobular lines, bronchiectasis, architectural distortion, and cysts [22,23,24] (Figure 9).

### 4.5. Radiation Pneumonitis

Radiation pneumonitis is an acute inflammatory reaction of the lung to radiation exposure, usually seen after thoracic radiotherapy. In the acute phase of radiation pneumonitis, swelling, vacuolation, and sloughing of both capillary endothelial and alveolar epithelial cells occur [25]. Proteinaceous fluid leaks from capillaries into the interstitium and then across the damaged alveolar epithelium into the alveolar spaces. Within alveolar spaces, hyaline membranes form from a combination of desquamated epithelial cells, fibrin-rich exudate, and macrophages [25].

CXR may show nonspecific airspace opacities and sometimes pleural effusions or atelectasis. Consolidation and GGOs are the main findings visualized on CT, often in association with geographic distribution, volume loss, and traction bronchiectasis. ILST is a less frequent finding on CT and can occur in both acute and chronic stages of radiation-induced lung injury.

### 4.6. Silicone Embolization

**Figure 10:**
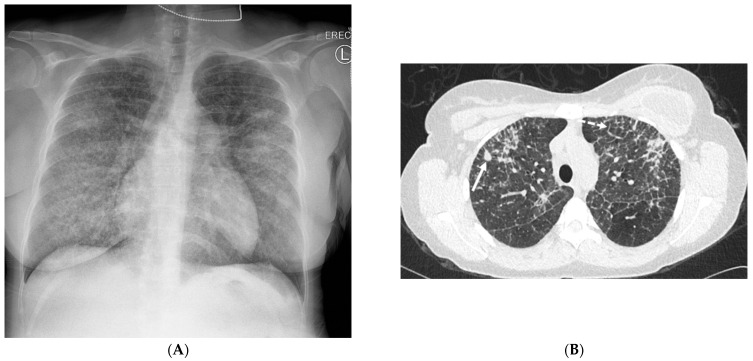

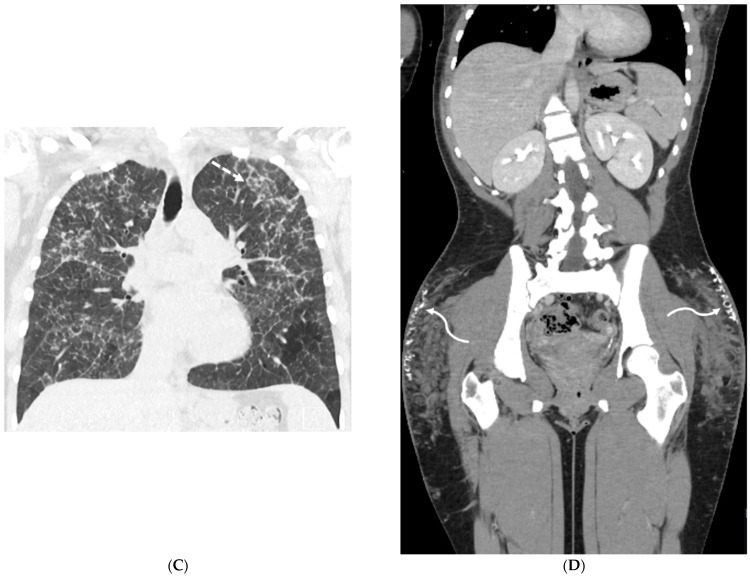
A 24-year-old female with a history of silicone injection of the buttocks presents with shortness of breath. A frontal chest radiograph (**A**) shows diffuse interstitial thickening. Axial (**B**) and coronal (**C**) chest CT images demonstrate upper- and mid-lung-zone-predominant perilymphatic nodularity (solid white arrow), interlobular (dashed white arrows) and intralobular nodular septal thickening, and irregular nodules associated with areas of interstitial thickening. A coronal pelvis CT image (**D**) demonstrates infiltrative soft tissue granulomas (curved white arrows), some calcified, with admixed areas of fat necrosis in bilateral gluteal subcutaneous fat.

The pathophysiology of silicone embolization is similar to that of fat embolization. The release of emboli leads to occlusion of the pulmonary microvasculature, triggering an inflammatory response. Generally, silicone embolization secondary to cosmetic procedures presents within the first two days following silicone injection. However, in some circumstances, the presentation may follow months after the injection. CT most often shows peripherally distributed GGOs associated with ILST [26] (Figure 10).

### 4.7. Pulmonary Vein Stenosis (PVS)

**Figure 11:**
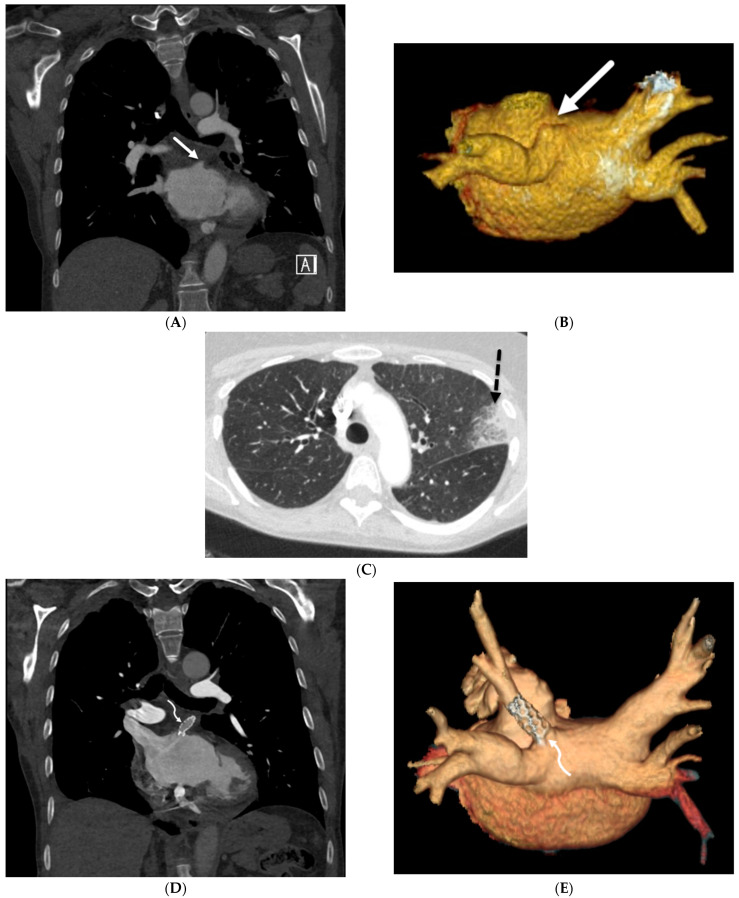
A 73-year-old female with atrial fibrillation refractory to medication post catheter ablation. Coronal chest CT and posterior view volume-rendered images of left atrium and pulmonary veins (**A**,**B**) demonstrate abrupt cut off of the left superior pulmonary vein (solid white arrow). An axial chest CT image (**C**) shows focal ground glass opacity and interlobular septal thickening (dashed black arrow) in the upper left lobe, representing pulmonary venous congestion and developing pulmonary venous infarct. Subsequent coronal chest CT (**D**) and posterior view volume-rendered images of the left atrium and pulmonary veins after stent placement (**E**) demonstrate the patency of the left superior pulmonary vein (curved white arrow).

The incidence of PVS after catheter ablation of the left atrium for atrial fibrillation ranges from 1% to 3%. Histopathologically, PVS manifests as a fibromuscular proliferation with occlusion of pulmonary veins. The pulmonary parenchyma is congested with interlobular and alveolar septal thickening, arteriopathic changes, and hemosiderosis [27]. CT in patients with PVS reveals bilateral scattered GGOs, diffuse ILST, and patchy consolidation (Figure 11).

## 5. Neoplasms

### 5.1. Invasive Lung Adenocarcinoma

**Figure 12:**
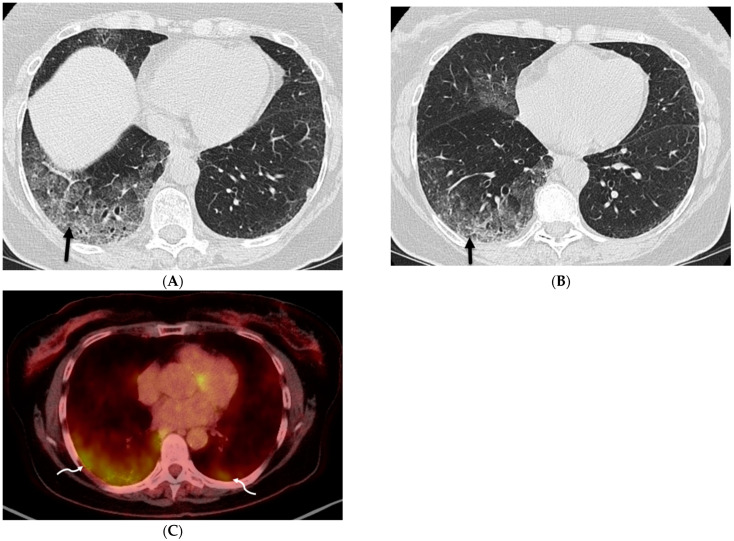
A 75-year-old former smoker presented with a history of chronic cough and was initially treated with antibiotics without improvement. Axial CT chest images obtained over a 4-month period (**A**,**B**) show worsening patchy ground glass opacity with areas of interlobular septal thickening (solid black arrow) in the right lower lobe. An axial fused FDG-PET/CT image (**C**) shows associated FDG uptake in lower lobes (curved white arrows), right greater than left, with a maximum standardized uptake value (SUV) of 5.4. Bronchoscopy confirmed the presence of multicentric invasive mucinous adenocarcinoma.

Lepidic-predominant adenocarcinoma (previously called non-mucinous bronchoalveolar lung carcinoma) arises along the bronchiolar and alveolar walls and is characterized by a lepidic growth pattern. In invasive mucinous adenocarcinoma (previously called mucinous bronchoalveolar lung carcinoma), the alveoli are filled with a low-density glycoprotein. The alveolar septa and underlying parenchymal architecture remain normal [28,29] (Figure 12).

### 5.2. Kaposi’s Sarcoma

**Figure 13:**
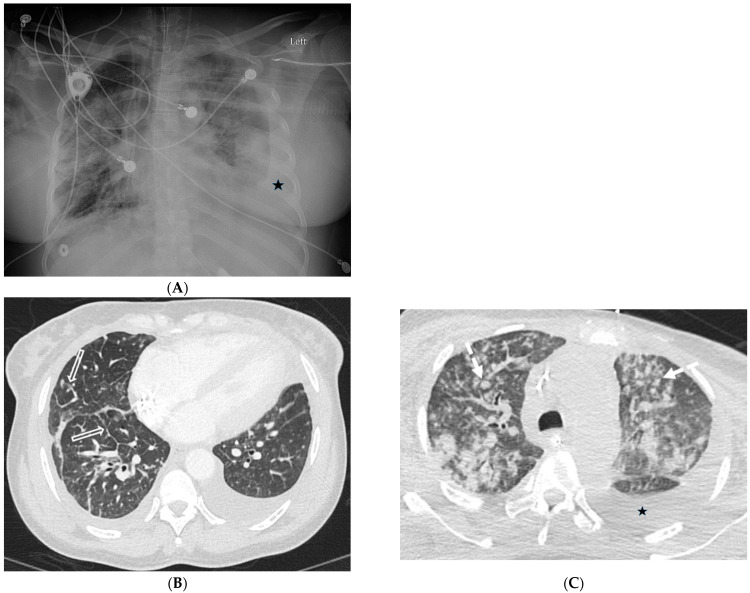
A 52-year-old female with advanced HIV infection and Kaposi’s sarcoma of the skin, lung, and gastrointestinal tract. A frontal chest radiograph (**A**) shows widespread nodules, consolidation, and bilateral pleural effusions. Axial chest CT images (**B**,**C**) demonstrate scattered ill-defined solid nodules (dashed white arrow), smooth interlobular septal thickening (open white arrows), and pleural effusions (stars).

A diagnosis of pulmonary involvement by Kaposi’s sarcoma usually can be made by a combination of clinical, radiographic, and laboratory findings, together with the results of bronchoscopy and transbronchial biopsy. CT scans commonly reveal peribronchovascular thickening and ILST, bilateral and symmetric ill-defined nodules in a peribronchovascular distribution, fissural nodularity, mediastinal lymphadenopathy, and pleural effusions. The presence of these findings in patients with AIDS is highly suggestive of Kaposi’s sarcoma [30] (Figure 13).

### 5.3. Lymphoma

The most common imaging patterns in primary lymphoproliferative lung diseases are consolidation, nodules, and masses (either solitary or multiple), usually with a random or perilymphatic distribution of spread [31]. ILST in lymphoma can be smooth or nodular. Secondary pulmonary lymphomas are known to present most commonly as bronchovascular or lymphangitic-like patterns with thickening of bronchovascular bundles and interlobular septa [32].

### 5.4. Leukemia

Nodules, GGOs, and consolidative opacities are also common with pulmonary involvement of leukemia. CT findings of leukemic pulmonary infiltration are nonspecific; however, leukemic cells have a tendency to involve the perilymphatic interstitium, producing smooth or nodular thickening of the bronchovascular bundles and interlobular septa. This occurs most commonly in the terminal stage of the disease but may also occur at other time points, including before treatment [33].

## 6. Depositional

### 6.1. Coal Worker’s Pneumoconiosis (CWP)

**Figure 14:**
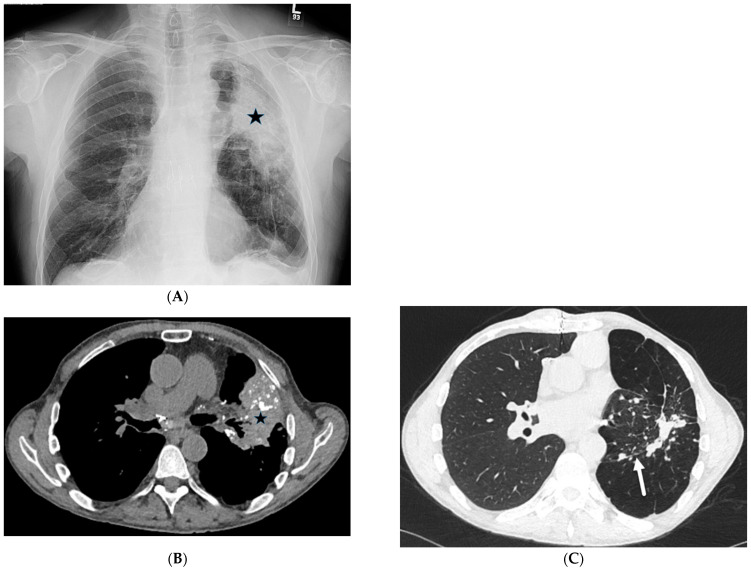
A 65-year-old male with a history of silicosis, coal worker’s pneumoconiosis, and pulmonary hypertension post right lung transplantation. A frontal chest radiograph (**A**) and axial chest CT images (**B**,**C**) demonstrate a partially calcified mass-like consolidative opacity in the left upper lobe (star) representing progressive massive fibrosis. Interlobular septal thickening (solid white arrow) is also noted in the left lower lobe on CT images.

CWP is common (occurring in 12–50%) in those exposed to coal dust for more than 20 years [34]. Upper lung zone-predominant diffuse, small, and round nodules are demonstrated on CXR and CT. Large conglomerate masses of fibrosis with architectural distortion may form with or without cavitation, usually in the posterior upper lung zones. ILST is a common finding in CWP and is caused by the accumulation of collagen and other proteins in the interlobular septa. Diffuse interlobular septal thickening can be seen in CWP (Figure 14).

### 6.2. Pulmonary Alveolar Proteinosis (PAP)

**Figure 15:**
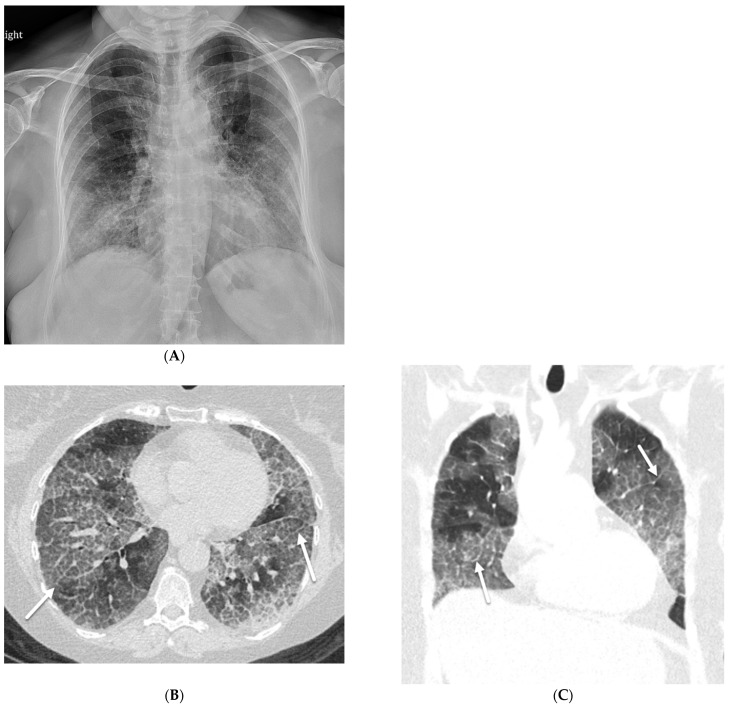
A 54-year-old female with a history of acute myeloid leukemia post allogenic bone marrow transplantation presents with pulmonary graft-versus-host disease. A frontal chest radiograph (**A**) demonstrates bilateral reticular opacities. Axial (**B**) and coronal (**C**) chest CT images reveal basilar predominant ground glass opacities with superimposed smooth interlobular septal thickening (solid white arrow) and intralobular reticulation, giving a crazy paving pattern. The patient was diagnosed with PAP after bronchoscopy.

Three subgroups of PAP are recognized: idiopathic, secondary, and congenital. Idiopathic PAP accounts for 90% of cases. Secondary PAP accounts for 5–10% of cases and is recognized in patients with industrial inhalational exposure to silica particles, cement dust, titanium dioxide, nitrogen dioxide, and fiberglass; underlying hematologic malignancy; or immunodeficiency disorders (including cytotoxic or immunosuppressive therapy and HIV infection). Congenital PAP is quite rare and accounts for 2% of cases and manifests in the neonatal period [35]. In PAP, ILST is likely due to edema, and GGO is caused by the filling of alveolar spaces with periodate–Schiff-positive proteinaceous material [36]. Although parenchymal involvement in PAP is usually bilateral and heterogeneous, with a typical mosaic pattern on CT scans, parenchymal involvement may be asymmetric on CXR in a small percentage of patients [37] (Figure 15).

### 6.3. Amyloidosis

Amyloidosis can be systemic or localized. Involvement of the lung is relatively common, although rarely symptomatic. Amyloidosis can appear in the lung in three different forms: nodular pulmonary amyloidosis, diffuse alveolar–septal amyloidosis, and tracheobronchial amyloidosis. Nodular pulmonary amyloidosis may manifest as solitary or multiple pulmonary nodules. Solitary tumor-like deposits (termed amyloidoma) are present in 60% of patients [38].

**Figure 16:**
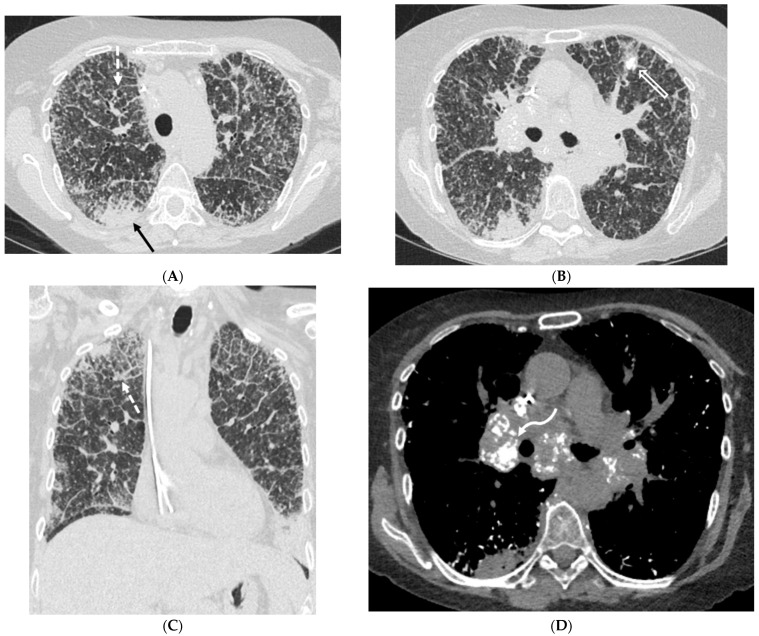
A 64-year-old female presenting with a history of amyloidosis. Axial CT images through the upper and mid thorax (**A**,**B**) and a coronal CT image (**C**) demonstrate diffuse nodular interlobular septal thickening (dashed white arrow) and innumerable micronodules throughout the lungs (open white arrow). Ill-defined areas of peripheral consolidation (solid black arrow) are also seen with a predilection for lower lung zones. An axial CT image displaying soft tissue windowing (**D**) demonstrates punctate calcifications within the lungs as well as partially calcified mediastinal and bilateral hilar lymphadenopathy (curved white arrow).

Multiple pulmonary nodules are common and may exhibit smooth, lobulated, or spiculated margins and occasional central or punctate calcification [39]. Rarely, lesions may cavitate. Diffuse alveolar septal pulmonary amyloidosis is characterized by well-defined 2–4 mm micronodules accompanied by reticular opacities, ILST, and confluent consolidations with basal and peripheral predominance on CT. Punctate lung calcifications, pleural effusions, and pleural thickening also may occur. Tracheobronchial amyloidosis can cause nodular lesions, circumferential wall thickening, and long-segment narrowing of the trachea and bronchi (Figure 16). Diagnosis through image evaluation alone is difficult. A biopsy is often required for definitive diagnosis.

### 6.4. Pulmonary Alveolar Microlithiasis

**Figure 17:**
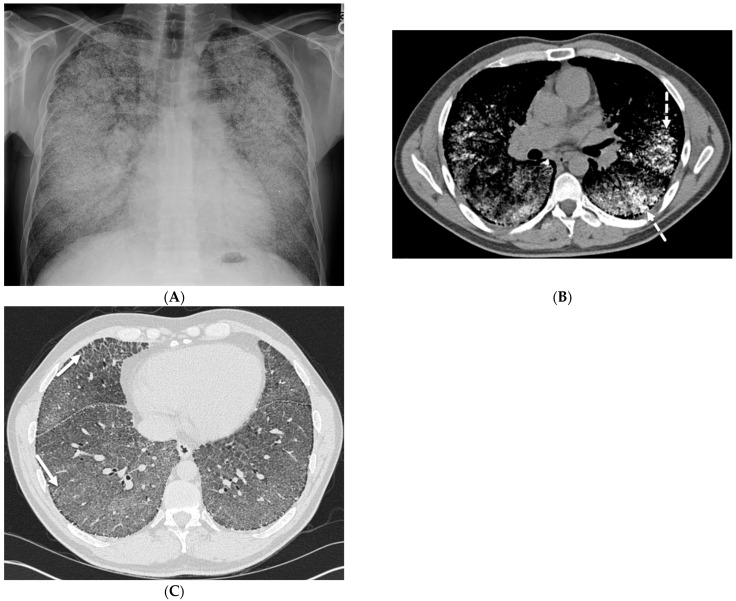
A 42-year-old male with a history of pulmonary alveolar microlithiasis. A frontal chest radiograph (**A**) and axial chest CT images (**B**,**C**) show a diffuse increase in pulmonary density with more confluent areas of calcification/ossification in both upper and mid lung zones (dashed white arrow). Interlobular septal thickening is noted in both lung bases (solid white arrow).

In pulmonary alveolar microlithiasis, CXR typically shows innumerable small, dense nodules, diffusely involving both lungs, predominantly in the lower zones. CT demonstrates widespread intra-alveolar microliths, diffuse GGOs, ILST, and subpleural sparing (also known as the black pleura sign), predominantly in the basal regions [40] (Figure 17).

## 7. Inherited

### 7.1. Yellow Nail Syndrome (YNS)

**Figure 18:**
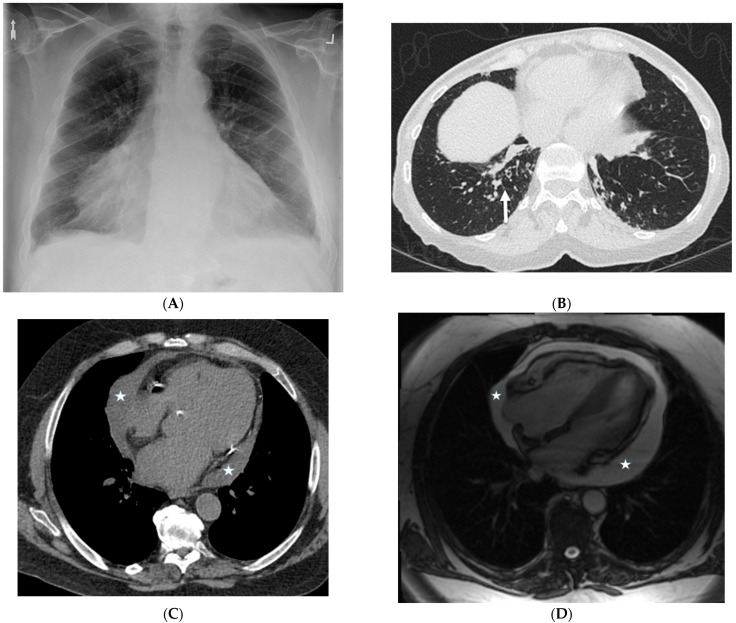
A 75-year-old man with a history of yellow nail syndrome presenting with chronic bronchitis and bronchiectasis. A frontal chest radiograph (**A**) shows an enlarged cardiac silhouette. An axial chest CT image (**B**) demonstrates cylindrical bronchiectasis with bronchial wall thickening and foci of peripheral mucoid impaction in lung bases and subtle interlobular septal thickening (solid white arrow) in the left lower lobe. An axial chest CT image (**C**) and axial bright-blood MR image (**D**) confirm moderate pericardial effusion (stars).

YNS is a rare clinical syndrome characterized by a triad of thick yellow nails, lymphedema, and respiratory disease. However, the complete triad is present only in 27–60% of patients, such that the presence of only two features may be considered sufficient for diagnosis. Various pulmonary manifestations of YNS are documented in the literature [41]. Woodfield et al. also demonstrated that YNS patients with bronchiectasis presented with ILST (32%), consolidation (21%), pleural effusions (20%), and focal tree-in-bud nodules [42] (Figure 18).

### 7.2. Pulmonary Capillary Hemangiomatosis (PCH) and Pulmonary Veno-Occlusive Disease (PVOD)

Pulmonary capillary hemangiomatosis (PCH) and pulmonary veno-occlusive disease (PVOD) were once considered separate entities, but are now thought to be variants of the same disease. They have many similarities in their clinical presentation, pathology, and response to treatment. They are rare causes of pulmonary hypertension with an estimated prevalence of one or two cases per 1 million inhabitants and an annual incidence rate of 0.1–0.5 per million [43]. The most common CT findings in PCH and PVOD include GGOs (often centrilobular), peribronchovascular thickening, ILST, and mediastinal lymphadenopathy. Associated findings of pulmonary hypertension, including dilation of the central pulmonary arteries, are also commonly seen.

### 7.3. Diffuse Pulmonary Lymphangiomatosis

**Figure 19:**
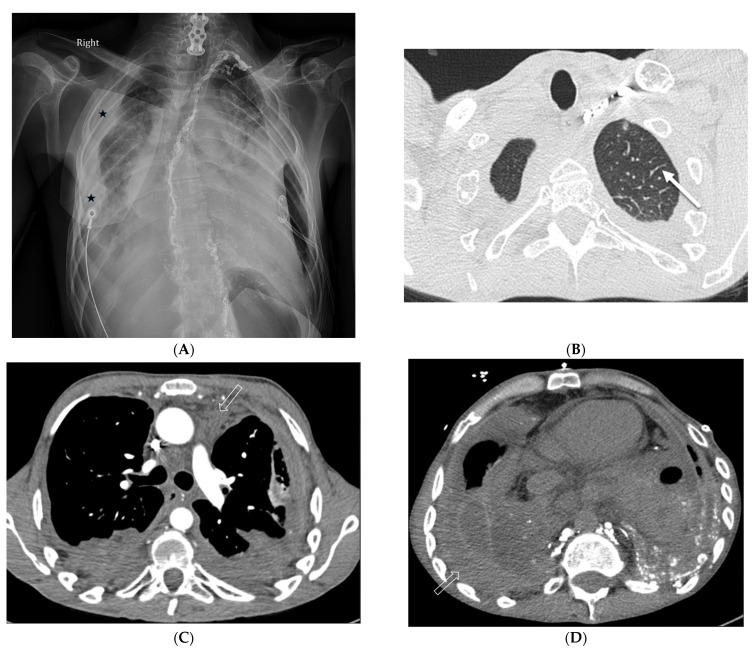

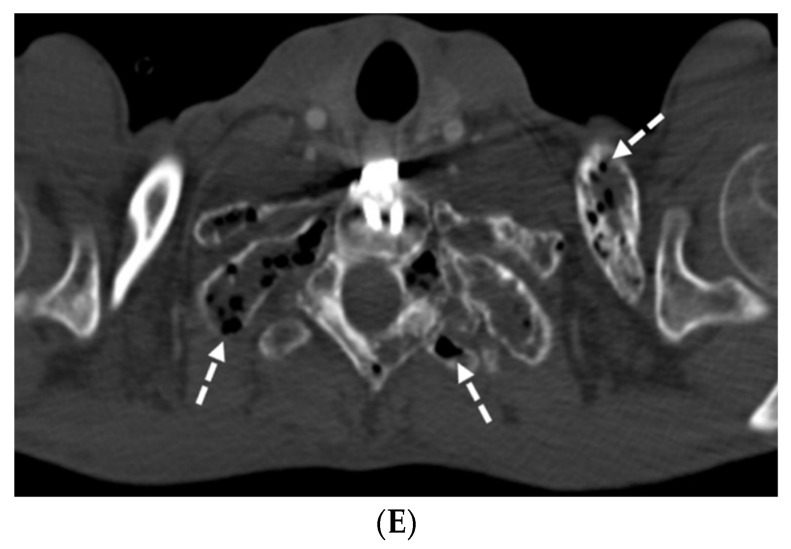
A 29-year-old male with diffuse pulmonary lymphangiomatosis presents with dyspnea. A frontal chest radiograph (**A**) and axial chest CT images (**B**–**E**) demonstrate smooth interlobular septal thickening (solid white arrow), multiloculated pleural effusions (stars on chest radiograph), diffuse infiltration of mediastinal and extrapleural fat (open white arrows), and lytic bone lesions (dashed white arrows). Multiple hyperattenuating punctate foci within pleural spaces represent residual lipiodol from prior lymphangiography (curved white arrow).

Diffuse pulmonary lymphangiomatosis (DPL) is a rare interstitial lung disease characterized by intrathoracic lymphatic system abnormalities, often with involvement of both lungs. Its common characteristics on CT include bilateral smooth ILST and peribronchovascular thickening, patchy GGOs, diffuse infiltration of the mediastinal and hilar fat, and bilateral pleural effusions. The manifestations of lymphangiomatosis that facilitate the diagnosis are bone lesions in conjunction with the chylothorax. Both CT and MRI can reveal lesions in the bone and soft tissue. On CT, soft tissue lesions in DPL typically appear as hypoattenuating masses with a lobulated or infiltrative margin. Bone lesions in DPL typically appear as hypoattenuating well-defined lesions with sclerotic borders [44] (Figure 19).

### 7.4. Congenital Pulmonary Lymphangiectasia

**Figure 20:**
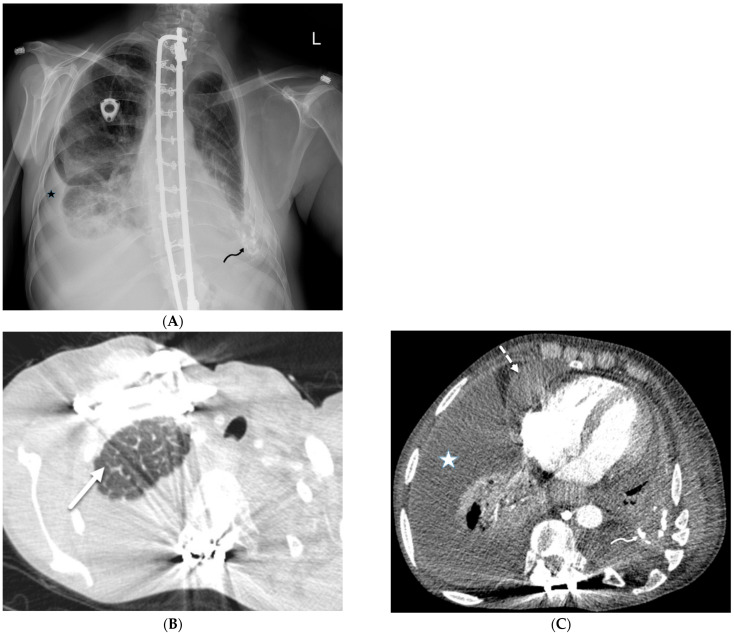
A 53-year-old female diagnosed with congenital pulmonary lymphangiectasia at birth. A frontal chest radiograph (**A**) and axial chest CT images (**B**,**C**) demonstrate smooth interlobular septal thickening (solid white arrow), bilateral pleural effusions (star), and pericardial effusion (dashed white arrow). High attenuation foci in the left pleural space (curved arrows) are related to prior pleurodesis.

Congenital pulmonary lymphangiectasia is a rare condition in which the lymph vessels in the lungs are abnormally dilated. It is seen almost exclusively in infancy and early childhood. CT demonstrates extensive bilateral ILST and peribronchovascular thickening, areas of GGO, and bilateral pleural effusions [45] (Figure 20). A diagnosis of congenital lymphangiectasia is usually made based on clinical symptoms, imaging findings, and lung biopsy.

### 7.5. Chronic Granulomatous Disease (CGD)

CGD is an X-linked recessive or autosomal recessive genetic disease. CGD may manifest as pneumonia, lymphadenopathy, liver abscess, soft tissue infection, osteomyelitis, suppurative arthritis, brain abscess, gastrointestinal infection, and organomegaly. Chronic pulmonary infection may be accompanied by pulmonary fibrosis, honeycomb lung, pulmonary artery hypertension, or pleural thickening [46].

### 7.6. Pulmonary Hyalinizing Granuloma (PHG)

Pulmonary hyalinizing granuloma is a very rare benign condition, which usually manifests as solitary and sometimes as multiple pulmonary nodules. The nodules can calcify but rarely cavitate. Up to 60% of PHG lesions show increased metabolic activity using ^18^F-fluorodeoxyglucose positron emission tomography/computed tomography (FDG-PET/CT). Nodular ILST along with extensive bilateral hilar and mediastinal lymphadenopathy can be seen. PHG can mimic other more common pulmonary disorders, such as neoplastic and granulomatous conditions. Therefore, histopathological examination is required to diagnose PHG and differentiate it from other nodular pulmonary disorders [47].

### 7.7. Coatomer Subunit Alpha (COPA) Syndrome

COPA syndrome results from autosomal dominant mutations affecting a narrow amino acid stretch in the COPA (coatomer subunit α) gene, encoding the COPα protein. The COPA gene is also associated with autoimmune diseases. The most prevalent imaging finding of COPA syndrome is diffuse lung disease, which can manifest as ground glass opacities, interstitial thickening, and airspace consolidation related to early childhood-onset recurrent pulmonary hemorrhage and lymphoid hyperplasia that may progress to pulmonary fibrosis. Less common CT findings include nodular opacities, bronchiectasis, hilar and mediastinal lymphadenopathy, and pleural effusions. Other imaging findings manifesting later in childhood or adolescence relate to arthritis and glomerulonephritis [48,49].

## 8. Miscellaneous

### 8.1. Diffuse Alveolar Hemorrhage (DAH)

**Figure 21:**
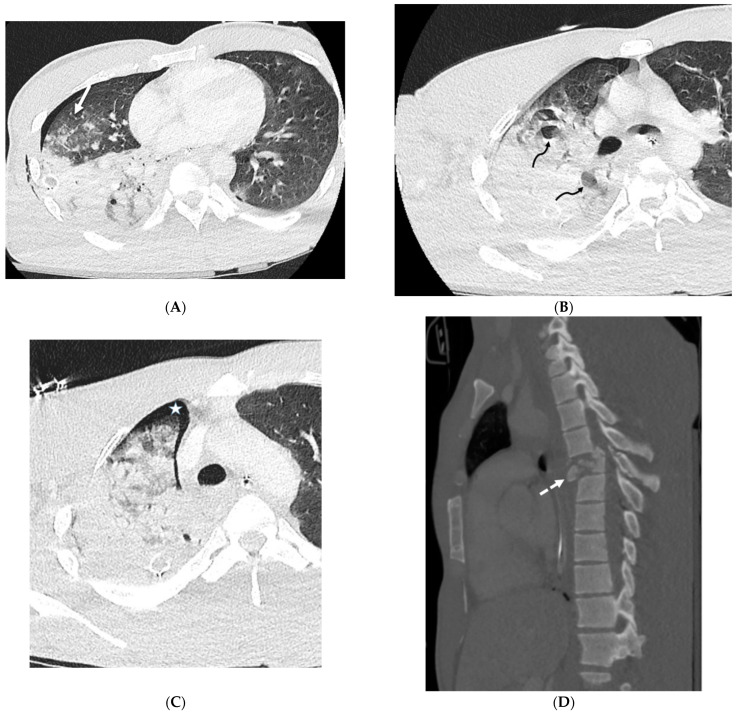
A 28-year-old male admitted after a motorcycle crash. Axial (**A**–**C**) and sagittal (**D**) chest CT images demonstrate interlobular septal thickening (solid white arrow) and airspace opacities in the right lung compatible with severe pulmonary hemorrhage and contusions. Pulmonary lacerations are seen as focal lucencies (curved black arrows) in the right upper and right lower lobes. Note small right pneumothorax (white star) and right chest wall subcutaneous emphysema as well. A sagittal chest CT image (D) shows an acute comminuted fracture of the T6 vertebra with retropulsion and spinal malalignment (dashed white arrow).

DAH can be caused by a variety of etiologies, including autoimmune disease, infection, and drug toxicity, and is characterized by bleeding into the alveoli. DAH presents on CT as multifocal GGOs with or without consolidation, often with poorly defined centrilobular nodules, sometimes with subpleural sparing [50]. Mild smooth ILST can be present days after the onset of DAH and is often associated with GGO, producing the crazy paving pattern (Figure 21). Treatment for DAH typically includes corticosteroids, immunosuppressants, and oxygen therapy.

### 8.2. Granulomatous–Lymphocytic Interstitial Lung Disease (GLILD)

**Figure 22:**
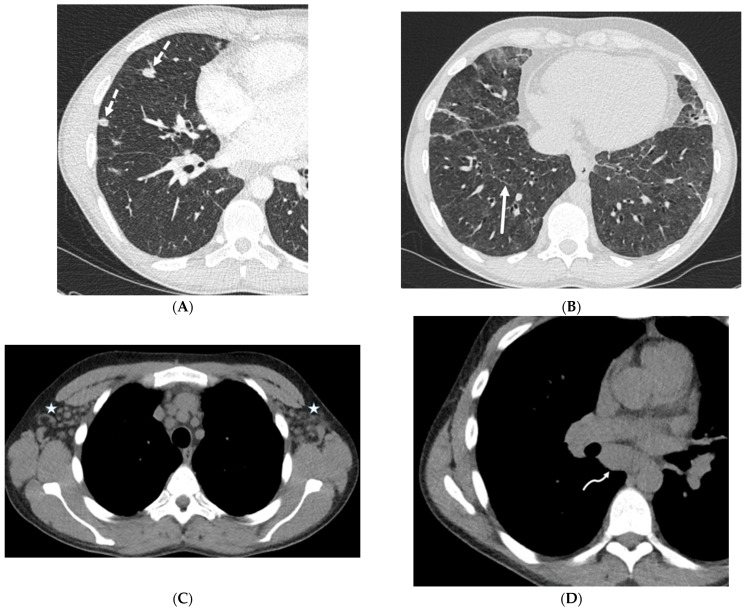
A 35-year-old male with a history of common variable immune deficiency (CVID) complicated by granulomatous–lymphocytic interstitial lung disease (GLILD). Axial chest CT images in lung windows (**A**,**B**) show extensive ground glass opacities in lungs with areas of interlobular septal thickening (solid white arrow) and discrete solid nodules (dashed white arrows). Axial chest CT images in soft tissue windows (**C**,**D**) show bilateral axillary (stars) and mediastinal (curved white arrow) lymphadenopathy.

GLILD is a rare disorder characterized by lymphocytic infiltration and the presence of granulomas in the lungs. It is also a complication of common variable immunodeficiency (CVID) [51]. The clinical features of GLILD range from major symptoms of breathlessness and cough to being asymptomatic. Diffuse pulmonary nodules surrounded by GGOs with lower lobe predominance, ILST predominantly in the mid to lower lung zones, lymphadenopathy, and a waxing and waning appearance of imaging findings over time may be seen in GLILD [52] (Figure 22).

### 8.3. Granulomatosis with Polyangiitis (GPA)

**Figure 23:**
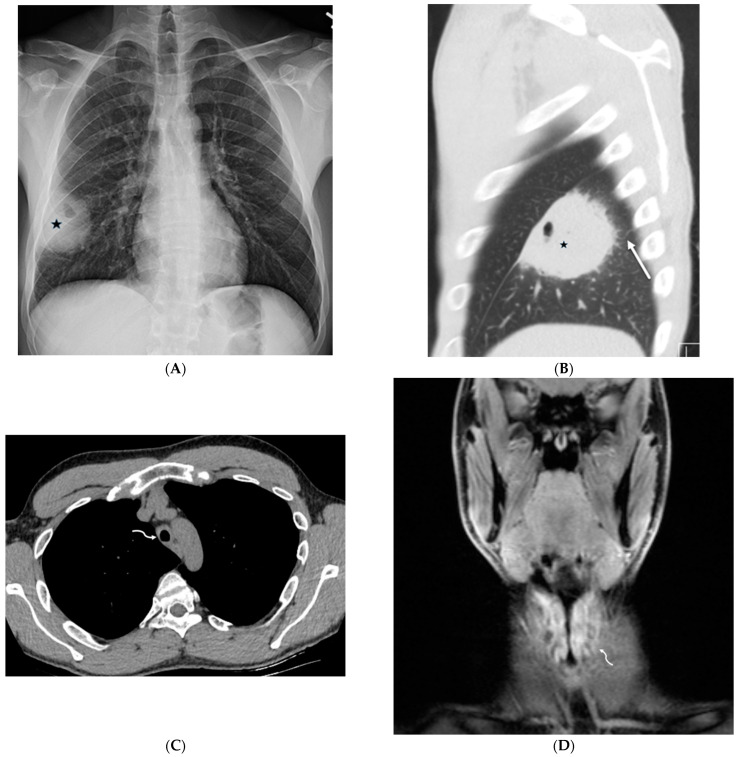
A 31-year-old male presenting with cough, intermittent nose bleeds, hoarseness, and a sensation of clogged ears. A frontal chest radiograph (**A**) demonstrates a cavitary mass (star) in the right lower lobe. A sagittal chest CT image (**B**) shows a cavitary mass (star) with surrounding interlobular septal thickening (solid white arrow). An axial chest CT image (**C**) and coronal neck fat-suppressed T1-weighted contrast-enhanced MR image (**D**) show marked luminal narrowing of trachea and subglottic larynx (curved white arrow) with circumferential soft tissue. A lung biopsy revealed multinucleated giant cells consistent with a diagnosis of granulomatosis with polyangiitis (GPA).

GPA is an autoimmune disease that affects blood vessels and organs, especially the lungs and kidneys. Lung consolidation and GGOs often occur in approximately 30% of patients with active GPA and are usually the result of hemorrhage. Pulmonary arteriolar involvement may cause mosaic attenuation or tree-in-bud opacities. ILST may also be seen due to lymphatic congestion or hemosiderin-laden macrophages [53] (Figure 23).

### 8.4. Erdheim Chester Disease (ECD)

**Figure 24:**
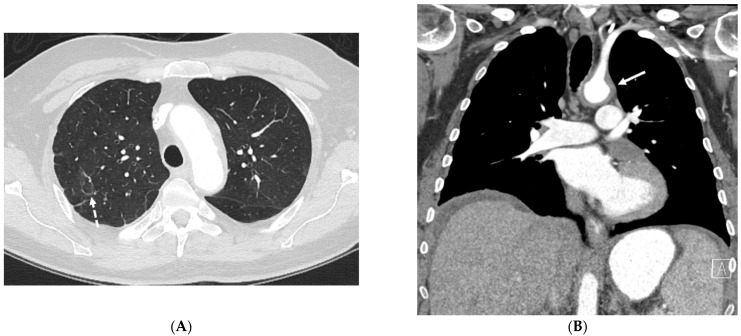

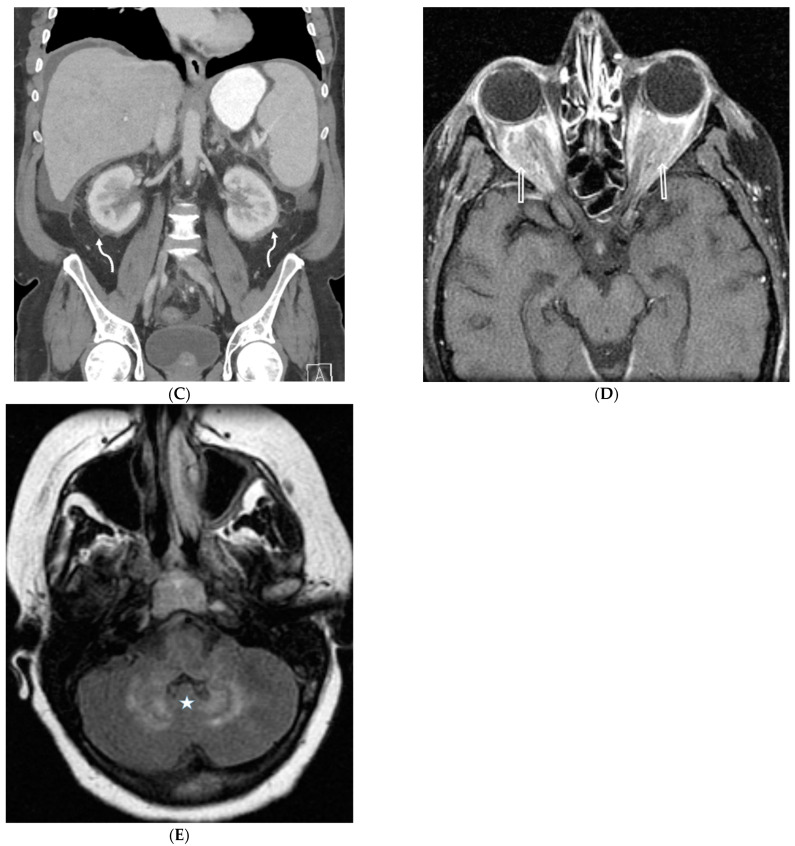
A 53-year-old male patient presents with shortness of breath and protruding eyes. Axial chest image (**A**) shows scattered foci of smooth interlobular septal thickening (dashed white arrow). A coronal chest CT image (**B**) demonstrates enhancing soft tissue surrounding the aortic arch (solid white arrow) consistent with aortitis. Coronal abdomen CT image (**C**) shows concentric perirenal soft tissue (curved white arrows) extending into the renal sinuses. An axial orbit fat-suppressed T1-weighted post-contrast MR image (**D**) shows extensive infiltration of intraconal spaces bilaterally with uniformly enhancing masses (open white arrows), along with an associated mass effect on both optic nerves and abnormal enhancement of both optic nerve sheaths. An axial head FLAIR MR image (**D**) shows foci of high signal intensity within the white matter of both cerebellar hemispheres (star) (**E**). A biopsy of perinephric soft tissue confirmed Erdheim Chester disease (ECD).

ECD is a rare disease that causes abnormal growth of histiocytes in various organs. The most common CT findings of ECD with pulmonary involvement include smooth ILST, centrilobular nodular opacities, fissural thickening, and pleural effusions [54]. Skeletal involvement may manifest as symmetric sclerosis in the diaphyses and metaphyses of the long bones, a coarsened trabecular pattern, and cortical thickening [55] (Figure 24).

### 8.5. Niemann–Pick Disease (NPD)

NPD is a lysosomal storage disorder that is subdivided into types A, B, and C based on the underlying metabolic deficiency. Pulmonary involvement occurs in all three types of NPD but is seen most frequently in type B. In types A and C, pulmonary involvement is less common and less severe. In type A, pulmonary involvement is usually seen in older children and adolescents. HRCT findings may include interstitial thickening, GGOs, and consolidation. In type B, the morphologic changes in CT include ILST, intralobular lines, and GGOs. Pulmonary nodules can also be seen in type B NPD [56]. In type C, pulmonary involvement is usually seen in adults. HRCT findings may include interstitial thickening, GGOs, consolidation, and bronchial wall thickening [57].

### 8.6. Gaucher Disease

Gaucher disease is a rare lysosomal storage disorder that can affect the lungs. The most common HRCT findings in Gaucher disease are interstitial thickening (both interlobular and intralobular), GGOs, consolidation, and bronchial wall thickening. Irregular interfaces at the pleural surfaces can also be seen. These findings are thought to be due to the infiltration of Gaucher cells into the lung parenchyma [58].

### 8.7. IgG4-Related Lung Disease

IgG4-related lung disease (IgG4-RLD) is a systemic fibroinflammatory condition that can mimic a variety of other lung diseases, including malignancy. The most common CT findings in IgG4-RLD are GGOs, peribronchovascular thickening, ILST, bronchiectasis, and a solitary large nodular lesion. Less common CT findings in IgG4-RLD include honeycombing, pleural thickening, and mediastinal fibrosis. IgG4-RLD often has a multifocal distribution [59].

**Algorithm or strategy for assessment of interlobular septal thickening (ILST):**

Step 1: Assess the characteristics of ILST:

- Nature of thickening: Smooth, nodular, or irregular
- Distribution: Diffuse, upper vs. mid vs. lower lung zone-predominant, central, peripheral, multifocal, or focal
- Symmetry: Symmetric or asymmetric

Step 2: Evaluate associated findings:

- Lung nodules/masses
- Ground glass opacity
- Consolidation
- Cavitation
- Cyst formation
- Airway involvement
- Lymphadenopathy
- Pneumothorax
- Pleural effusion
- Extrathoracic features

Step 3: Consider the clinical context:

- Symptoms and signs
- Past medical history
- Risk factors

Step 4: Narrow the differential diagnosis: Based on the combination of ILST characteristics, associated findings, and clinical context, develop a shortlist of potential causes for ILST.
Step 5: Utilize additional investigations (if necessary): Depending on the differential diagnosis, further investigations like pulmonary function testing, laboratory testing, bronchoscopy, or tissue sampling may be considered to establish a definitive diagnosis.

## 9. Conclusions

Interlobular septal thickening (ILST) is a frequently encountered finding on CT scans in a variety of lung disorders. While pulmonary edema and lymphangitic spread of tumors are common causes of ILST, a broad spectrum of less common underlying etiologies also exist. Therefore, consistent with prior literature, we emphasize the importance of a detailed analysis of ILST characteristics and accompanying pulmonary and extrapulmonary features to refine the differential diagnosis. This systematic approach is crucial for achieving a timely and accurate diagnosis.

**Essentials:**

1. Pulmonary edema and lymphangitic spread of tumors are the most commonly encountered causes of ILST.
2. This article reviews the spectrum of uncommon entities that present with thickening of the interlobular septa and establishes a practical approach for obtaining a differential diagnosis.
3. Detailed image analysis can help us to distinguish between conditions with overlapping imaging characteristics.

**Summary Statement:**

Careful assessment of the appearance and distribution of ILST, as well as of other pulmonary and extrapulmonary findings, is useful to obtain a differential diagnosis of potential underlying causes.

## Figures and Tables

**Table 1:** Common and uncommon causes of interlobular septal thickening.

Infection	Viral pneumonia including COVID-19 pneumonia Pneumocystis jiroveci pneumonia (PJP)
Inhalational	Silicosis Coal worker’s pneumoconiosis (CWP) Asbestosis
Interstitial lung disease	Nonspecific interstitial pneumonia (NSIP) Usual interstitial pneumonia (UIP) Lymphoid interstitial pneumonia (LIP) Chronic hypersensitivity pneumonitis (HP)
Granulomatous diseases	Sarcoidosis Erdheim Chester disease (ECD)
Neoplasia	Leukemia Lymphoma Kaposi’s sarcoma
Depositional	Pulmonary alveolar proteinosis (PAP) Amyloidosis
Iatrogenic	Drug toxicity Radiation therapy Silicone embolization Lipoid pneumonia E-cigarette or vaping use-associated lung injury (EVALI)
Inherited	Chronic granulomatous disease Yellow nail syndrome (YNS) Pulmonary capillary hemangiomatosis (PCH) Pulmonary lymphangiomatosis Niemann–Pick disease Gaucher disease Coatomer subunit alpha (COPA) syndrome

**Table 2:** Causes of smooth, nodular, and irregular interlobular septal thickening.

Smooth 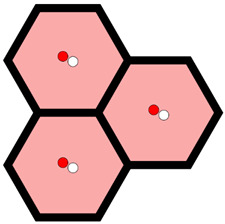	Infectious: Viral pneumonia including COVID-19 pneumonia, Pneumocystis jiroveci pneumonia (PJP) Miscellaneous: Pulmonary edema, pulmonary hemorrhage Inherited: Yellow nail syndrome (YNS), pulmonary capillary hemangiomatosis (PCH), pulmonary veno-occlusive disease (PVOD), pulmonary lymphangiomatosis, coatomer subunit alpha (COPA) syndrome Depositional: Pulmonary alveolar proteinosis (PAP) Iatrogenic: Drug toxicity, radiation pneumonitis, lipoid pneumonia, e-cigarette or vaping use-associated lung injury (EVALI) Granulomatous: Erdheim Chester disease (ECD)
Nodular 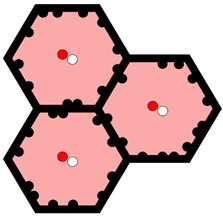	Neoplasia: Leukemia, lymphoma, Kaposi’s sarcoma, lymphangitic carcinomatosis Interstitial lung diseases: Nonspecific interstitial pneumonia (NSIP), usual interstitial pneumonia (UIP), lymphoid interstitial pneumonia (LIP) Exposures: Silicosis, coal worker’s pneumoconiosis (CWP), asbestosis Inherited: Chronic granulomatous disease, Niemann–Pick disease Depositional: Amyloidosis Iatrogenic: Silicone embolization Granulomatous diseases: Sarcoidosis Infiltrative: IgG4-related lung disease (RLD)
Irregular 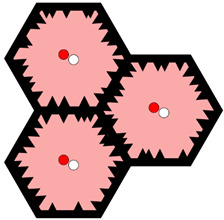	Granulomatous diseases: Sarcoidosis Interstitial lung diseases: Usual interstitial pneumonia (UIP) Inhalational: Asbestosis

**Table 3:** Causes of interlobular septal thickening and their thoracic imaging features.

Diagnosis	Type and Location of ILST	Nod	GGO	Con	CAV	Cy	LAN	Fib	PTX	PEF
Pneumocystis jiroveci pneumonia (PJP)	smooth, perihilar, and lower lobe predominance	−	++	−	−	+	−	−	+	−
Sarcoidosis	nodular	+	+	+	+/−	+/−	++	++	+/−	+
Coal worker’s pneumoconiosis (CWP)	nodular, diffuse	+	+/−	+	+/−	−	+	+	−	−
Nonspecific interstitial pneumonia (NSIP)	irregular	−	++	−	−	−	+	+	+	−
Lymphoid interstitial pneumonia (LIP)	nodular	+	+	−	−	+	+	−	+/−	−
Lipoid pneumonia	smooth	+	+	++	−	−	−	−	−	−
Drug-induced toxicity	smooth	+	+	+	−	−	−	+	−	−
Radiation pneumonitis		−	+	+	+	−	−	+	−	+
Electronic cigarette or vaping product use-associated lung injury (EVALI)	smooth	+	+	+	−	−	−	−	+	+/−
Silicone embolization	diffuse	+	+	+	−	−	−	−	−	+
Pulmonary alveolar proteinosis (PAP)	smooth	+	++	+	−	−	−	+/−	−	−
Lymphoma	nodular	++	+/−	+	+/−	−	++	−	−	+
Kaposi’s sarcoma	nodular	+	+	+	−	−	+	−	−	+
Pulmonary amyloidosis	nodular	+	+	+	+	+	+	+	+	+
Pulmonary hyalinizing granuloma (PHG)	nodular	+	−	−	−	−	+	−	−	−
Chronic granulomatous disease	smooth	+	+	+	+	−	+	+	−	+
Erdheim Chester disease (ECD)	smooth, diffuse, sometimes nodular	+	+	+	−	+	−	−	−	+
Niemann–Pick disease	smooth, lower lobe predominance	−	++	−	−	−	−	−	−	−
Gaucher disease	smooth	+	++	−	−	−	−	−	−	−
IgG4-RLD	nodular	+	+	+	−	−	++	+	−	−
Yellow nail syndrome	smooth	−	+	−	−	−	−	−	−	++
Diffuse pulmonary lymphangiomatosis	smooth, diffuse	−	+	−	−	−	++	−	−	+
Pulmonary capillary hemangiomatosis (PCH)	smooth	+	+	−	−	−	+/−	−	−	+/−
Coatomer subunit alpha (COPA) syndrome	smooth, mid, and lower lung zone predominance	−	+	−	−	++	−	−	−	−

Note: Plus signs indicate the relative frequency of the findings from low (+) to high (++); − = not seen; +/− = may or may not be seen. Nod = nodule; GGO = ground glass opacity; CON = consolidation; CAV = cavitation; Cy = Cyst; LAN = lymphadenopathy; Fib = fibrosis; PTX = pneumothorax; PEF = pleural effusion; ILST = interlobular septal thickening.

**Table 4:** Causes of interlobular septal thickening and associated distinctive clinical features and extrapulmonary findings.

Diagnosis	Associated Distinctive Clinical Features	Associated Extrapulmonary Findings
Pneumocystis jiroveci pneumonia (PJP)	Immunosuppression; CD4 count, <200 cells/mm	Low-attenuation splenic lesions that may calcify; punctate calcifications in the liver, renal cortices, and adrenal glands; calcification of lymph nodes; and pleural and peritoneal effusions with subsequent calcifications
Sarcoidosis	African Americans and Northern European Caucasians	Cardiomegaly, pericardial effusion, ventricular aneurysm, focal myocardial thickening, cystic bone lesions, lace-like pattern of lytic change in the metaphyses of long bones, neurosarcoidosis
Coal worker’s pneumoconiosis (CWP)	Exposure to coal dust free of silica	No specific findings
Nonspecific interstitial pneumonia (NSIP)	Often history of connective tissue disorder or other predisposing condition	Dilated patulous esophagus often containing fluid or debris in scleroderma; pleural thickening or effusion in rheumatoid arthritis; pleural and pericardial effusions in systemic lupus erythematosus; and pleural effusions in mixed connective tissue disease
Lymphoid interstitial pneumonia (LIP)	May be associated with HIV infection and Sjogren’s syndrome	Lymphadenopathy
Lipoid pneumonia	Prior history of mineral oil ingestion	No specific findings
Drug-induced toxicity	History of recent drug exposure	Neurological—encephalopathies, meningitis, Guillan Barré syndrome, and hypophysitis Gastrointestinal—esophagitis, gastritis, enteritis, colitis, ulcer formation, angioedema, pneumatosis, bowel perforation Liver—hepatic steatosis, hepatitis, cirrhosis, Budd–Chiari syndrome, sinusoidal obstruction syndrome, hepatomegaly, periportal edema, periportal lymphadenopathy Pancreaticobiliary—acute pancreatitis, cholangitis
Radiation pneumonitis	History of prior radiation therapy	Lymphedema, calcification of lymph nodes, pericardial effusion, pericardial fibrosis, brachial plexus injury, osteitis, pathological fractures
Electronic cigarette or vaping product use-associated lung injury (EVALI)	History of vaping (whether new or increased use)	Pneumomediastinum, pleural effusion
Silicone embolization	History of illegal cosmetic injection of liquid silicone	Multiple subcutaneous soft tissue attenuation nodules, sometimes with peripheral calcification or surrounding fat stranding
Pulmonary alveolar proteinosis (PAP)	Tobacco use; subacute symptom onset; history of prior episodes	May occur in association with malignancies, whether pulmonary or extrapulmonary, most commonly hematologic
Lymphoma	Patients with autoimmune diseases such as rheumatoid arthritis, Sjögren’s syndrome, and systemic lupus erythematosus are at increased risk of malignant lymphomas	Hodgkin lymphoma more commonly involves only lymph nodes, whereas non-Hodgkin lymphomas more commonly involve extranodal structures and can involve any organ
Kaposi sarcoma	Associated with HIV infection	Cutaneous lesions, mucosal involvement, lytic bone lesions, nodular lesions in the liver and spleen
Pulmonary amyloidosis	Secondary amyloidosis accompanying a chronic inflammatory suppurative process can be related to tuberculosis, bronchiectasis, rheumatoid arthritis, or osteomyelitis.	Multiple intracranial hemorrhages, hepatosplenomegaly, global subendocardial or transmural late gadolinium enhancement on cardiac MRI
Pulmonary hyalinizing granuloma (PHG)	Individual predisposed to marked scar formation	No specific findings
Chronic granulomatous disease	Two-thirds of individuals have the X-linked recessive form of the disorder.	Hepatosplenomegaly, liver abscess, osteomyelitis

## Data Availability

Not applicable.

## Abbreviations

Interlobular septal thickening (ILST), interstitial lung disease (ILD), ground glass opacity (GGO), e-cigarette or vaping use-associated lung injury (EVALI), diffuse alveolar hemorrhage (DAH), granulomatous–lymphocytic interstitial lung disease (GLILD), granulomatosis with polyangiitis (GPA), congestive heart failure (CHF), coal worker’s pneumoconiosis (CWP), idiopathic pulmonary fibrosis (IPF), nonspecific interstitial pneumonia (NSIP), usual interstitial pneumonia (UIP), lymphoid interstitial pneumonia (LIP), acute interstitial pneumonia (AIP), hypersensitivity pneumonitis (HP), pulmonary alveolar proteinosis (PAP), Erdheim Chester disease (ECD), yellow nail syndrome (YNS), pulmonary capillary hemangiomatosis and pulmonary veno-occlusive disease (PCH-PVOD), coatomer subunit alpha (COPA) syndrome, pulmonary hyalinizing granuloma (PHG), IgG4-related lung disease (IgG4-RLD).
